# The PEAPOD repressor complex in Arabidopsis stomatal development

**DOI:** 10.3389/fpls.2025.1641102

**Published:** 2025-07-23

**Authors:** Josué Saiz-Pérez, Carmen Fenoll, Montaña Mena

**Affiliations:** Facultad de Ciencias Ambientales y Bioquímica, Universidad de Castilla-La Mancha, Toledo, Spain

**Keywords:** stomatal development, asymmetric cell division, PEAPOD, leaf development, light signaling, cell cycle

## Abstract

Stomata comprise two guard cells that function as microscopic valves in the plant epidermis, connecting mesophyll interstices to the atmosphere. Stomata regulate gas exchange and evapotranspiration, directly impacting photosynthesis and leaf temperature regulation, and their function is thus crucial for plant adaptability and fitness. In Arabidopsis, stomatal development is primarily driven by three basic helix-loop-helix transcription factors: SPEECHLESS (SPCH), MUTE, and FAMA, and occurs within the broader context of leaf development. During leaf development, a characteristic division-to-differentiation transition zone, marked by the first cell cycle arrest front (1^st^ AF), progresses from the apex to the base of the leaf blade. The repeated division of meristemoids (M), self-renewing cells of stomatal lineages, is not halted during 1^st^ AF, requiring a second arrest front, which is associated with activity of the PEAPOD (PPD) proteins, PEAPOD1 (PPD1) and PEAPOD2 (PPD2), which form a transcriptional repressor complex that halts M stem cell-like activity; however, the relationship between PPDs and stomatal development has not been fully elucidated. Here, we review data on PPD-mediated regulation of light signaling and the cell cycle and the influence of these factors on stomatal development.

## 1 Introduction

Multicellular organisms are generated through a spectrum of timely cell fate decisions, comprising both division and differentiation, that occur within developmental windows, ensuring functional organ development in mature organisms. For example, the plant leaf is an organ generated by an intricate molecular interplay that determines its shape, size, symmetry, and dorsoventrality. Investigation of the specific genetic determinants influencing leaf blade development (Kierzkowski et al., 2019) has identified gradients of morphogens (Ten Tusscher, 2020), known as mobile growth factors (MGFs) that, in combination with two cell cycle arrest fronts, drive a switch from leaf cell proliferation to differentiation (White, 2006; Kazama et al., 2010). Leaf development involves timely transcriptional regulation of a plethora of master regulatory genes in overlapping domains, to establish a specific pre-determined pattern (Vanhaeren et al., 2014, 2015; Castelán-Muñoz et al., 2019; Qiu et al., 2024).

Stomata are microscopic valve structures in the leaf epidermis comprising two guard cells (GCs) arising from meristemoids (Ms), cells with stem cell-like activity that contribute to leaf development. The function of stomata is to dynamically connect plant mesophyll to the atmosphere, and is indispensable for plant viability (Dittberner et al., 2018; Driesen et al., 2020). The opening and closing of stomata, along with their abundance and distribution, are regulated by physiological and environmental cues that modify gas exchange and evapotranspiration, directly influencing photosynthesis and leaf temperature regulation (Doheny-Adams et al., 2012; de Marcos et al., 2015; Pérez-Bueno et al., 2022). In addition to stomatal dynamics and function, a network of stomatal development genes controls their abundance and distribution. In *Arabidopsis thaliana* (Arabidopsis) stomatal development occurs through serial stereotypical division-differentiation events involving well-established cell types that are broadly distributed throughout the leaf epidermis (Bergmann and Sack, 2007), and is primarily driven by three basic helix-loop-helix transcription factors: SPEECHLESS (SPCH) (MacAlister et al., 2007), MUTE (Pillitteri et al., 2007), and FAMA (Ohashi-Ito and Bergmann, 2006; Jordan et al., 2015). These transcription factors require heterodimerization with SCREAM/SCREAM2 (SCRMs) to regulate stomatal-related genes (Kanaoka et al., 2008), and alteration of these key regulators modifies cell number and organ size, underlining their relevance in leaf development (Pérez-Bueno et al., 2022). In addition, the stomatal gene network comprises several positive and negative regulators, whose modulation alters stomata quantity and distribution (Saiz-Pérez et al., 2024). In the context of leaf development, Ms stem cell-like activity directly impinge on stomatal abundance in the mature organ, and therefore on physiological status (Dow et al., 2014), resulting in an adaptative trait with a strong genetic basis, as revealed by the broad natural variability observed in Arabidopsis (Delgado et al., 2011; Dittberner et al., 2018; Delgado et al., 2019).

Stomata are generated by self-renewing activity of Ms, broadly dispersed stomatal precursor cells which retain the capacity to divide several times without changes in cell-type fate, and are pivotal to epidermal development (Smit and Bergmann, 2023). Among the main characteristics of Ms is asymmetric cell division (ACD). Ms are generated from a meristemoid mother cell (MMCs) through an entry ACD that generates a M and a stomatal lineage ground cell (SLGC). Amplifying divisions allow M self-renewal, while generating neighboring SLGCs which subsequently differentiate into pavement cells (PCs) (Shpak et al., 2005). Alternatively, spacing ACDs generate satellite Ms from SLGCs, maintaining a one-cell spacing rule, which ensures proper stomata function (Hara et al., 2007; Dow et al., 2014). This remarkable ACD capacity generates around 65% to 82% of all leaf epidermal cells (Geisler et al., 2000). Interestingly, amplifying M division is absent in monocot plants, due to a lack of M self-renewal capacity (Vatén and Bergmann, 2012).

In Arabidopsis, leaf growth involves the interconnection of proliferation and expansion of leaf primordia cells (Asl et al., 2011), commencing with a group of founder cells flanking the shoot apical meristem (SAM) (Reinhardt et al., 2000; Kalve et al., 2014), which undergo profuse division, directly increasing cell numbers to form the mature organ; this proliferation phase is restricted to a specific time frame. Upon reaching a threshold, active mitotic cells exit the cell cycle and start to expand, while some PCs continue to increase in size through endocycles (Breuer et al., 2010; Magyar et al., 2012). Hence, several cellular processes determine final leaf shape and size, as follows: initial number of founder cells derived from the SAM; cell division rate; timing of cell division span and cell expansion; and extent of M proliferation (Hepworth et al., 2018). Alteration of any of these elements will modify mature leaf size (Gonzalez et al., 2012).

A crucial element controlling the division to differentiation switch in leaf blades is the 1^st^ cell cycle arrest front (1^st^ AF), which progresses to the base of the leaf blade from the apical region via MGFs (Kazama et al., 2010; Andriankaja et al., 2012). In Arabidopsis, stomatal proliferation and commitment also follow a tip-to-base direction; however, 1^st^ AF does not stop self-renewing capacity of Ms, which requires the action of a 2^nd^ arrest front (2^nd^AF) that has been linked to PEAPOD (PPD) proteins. There are two PPD paralogs, PEAPOD1 (PPD1) and PEAPOD2 (PPD2), which halt stomatal lineage stem cell-like activity, causing a switch of Ms from proliferative to committed states (White, 2006).

The aim of this review was to integrate knowledge of PPDs functions, focusing on PPDs modulation of Ms self-renewal activity. We explore the relationship of PPDs with stomatal development, light-mediated regulation, and the cell cycle machinery, whilst placing them into molecular context.

## 2 Stomatal lineage meristemoids display stem cell-like activity after 1^st^ AF

**Figure 1:**
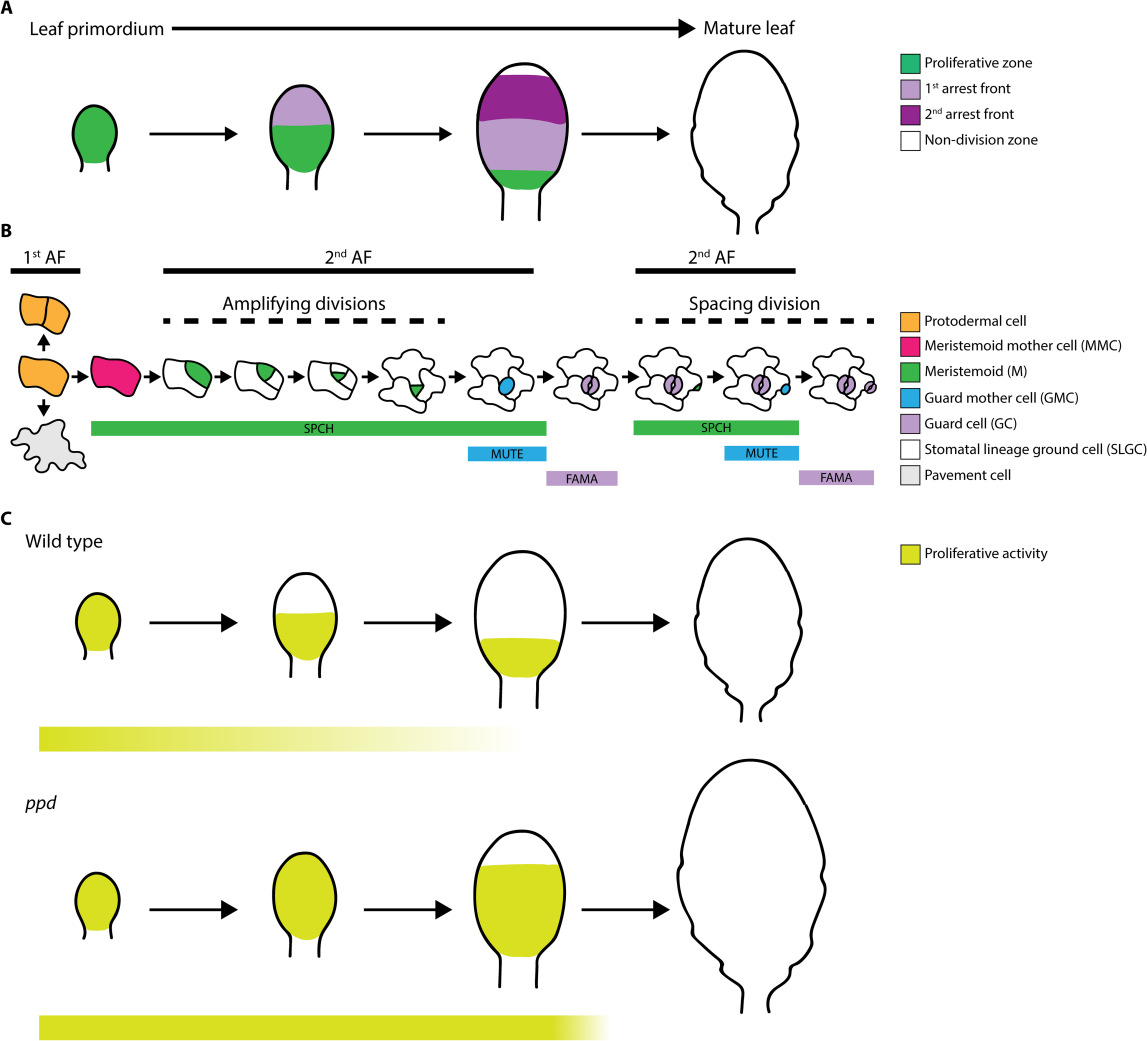
**(A)** Schematic overview of the proliferative zone, 1^st^ cell cycle arrest front (1^st^ arrest front), 2^nd^ cell cycle arrest front (2^nd^ arrest front), and non-division zone during development from leaf primordium to mature leaf in Arabidopsis. **(B)** Stomatal lineage cell types found in leaf epidermis: protodermal cell, meristemoid mother cell (MMC), meristemoid (M), guard mother cell (GMC) and guard cell (GC), stomatal lineage ground cell (SLGC) and pavement cell. Among them, only M exhibit proliferative activity. Cell types where 1^st^ arrest front (1^st^ AF) and 2^nd^ arrest front (2^nd^ AF) act are depicted with bold lines. Amplifying and spacing divisions in the stomatal lineages are indicated with dashed lines. **(C)** Comparison of proliferative activity zones during leaf development in wild type (above) and *ppd* (below) mutant plants. Extended M proliferative activity in *ppd* mutants is depicted in pale green bars below. Note that *ppd* mutation results in bigger leaves.

Progression of 1^st^ AF in developing leaves causes transition from cell proliferation to differentiation, involving post-mitotic cell expansion directly related to maturation. There is dynamic interplay between 1^st^ AF and MGF gradients, where maximum MGF concentrations occur at the leaf base, although AF exhibits different thresholds for MGFs depending on spatial coordinates, causing cell division to stop based on organ dynamics and developmental stage (Kazama et al., 2010). *CYCB1;1* reporters, which mark a linear border of actively dividing cells, have been used as evidence of 1^st^ AF progression in leaf primordia (Kazama et al., 2010; Baekelandt et al., 2018). In contrast to SAM and root apical meristem (RAM), which divide continuously, maintaining constant meristem size and constituent cell number (Miwa et al., 2009), the active dividing regions change dynamically in Arabidopsis leaves. Hence, unlike the constant activity of SAM and RAM, leaf blade 1^st^ AF does not progress uniformly. During a specific period, 1^st^ AF imposes a non-dividing zone in more distal regions of the leaf blade, while remaining unaltered close to blade base, generating a proliferation zone and consequent leaf morphogenesis; however, while 1^st^ AF halts protodermal cells division, it does not restrict ACD activity of Ms. Prevention of stomatal lineage cell ACD activity requires a 2^nd^ AF, driven by PPDs (**Figure 1A**), which also modify the shape of the 1^st^ AF (Baekelandt et al., 2018).

## 3 Proliferative capacity of meristemoids

Stomatal lineages begin with acquisition of meristemoid mother cell (MMC) identity by a protodermal cell via SPCH expression. SPCH activity triggers an entry ACD, generating a M retaining the ability to divide asymmetrically through reiterative amplifying divisions (**Figure 1B**), a process known as the proliferative stage of stomatal lineage, which relies on M self-renewal capacity (Han et al., 2022); whereas in SLGCs, SPCH activity causes spacing divisions generating satellite Ms (**Figure 1B**). Ms can go through up to three amplifying ACDs before differentiating into guard mother cells (GMCs) via activity of MUTE and its downstream genes, which orchestrate a symmetrical cell division to generate the GCs comprising mature functional stomata (Kim and Torii, 2024). Subsequently, FAMA prevents further GC divisions (Hachez et al., 2011). Unlike Ms, GMCs and GCs do not exhibit proliferative activity; nevertheless, SPCH activity is not restricted to M stages, and overlaps with MUTE expression domains (Lopez-Anido et al., 2021).

The prolonged stage of proliferative activity involving meristemoid ACDs is a key mechanism regulating leaf development. To date, the only molecules associated with this predefined state controlling ACDs are PPDs. Deletion of genes encoding PPDs (i.e., *ppd* mutants) results in larger leaves, due to PPDs role in repressing cell proliferation across leaf tissues, including mesophyll cells and stomatal lineage cells (**Figure 1C**; White, 2006). Similarly, artificial microRNA targeting genes encoding both PPDs (i.e., *ami-ppd*) led to similar phenotypes (Gonzalez et al., 2015). Nevertheless, although transcripts encoding SPCH, MUTE, TMM, and POLAR are up-regulated in *ppd* mutants, none of these genes are direct targets of PPD2 (Gonzalez et al., 2015), indicating that PPDs indirectly modulate stomatal development.

## 4 PEAPOD complex function in stomatal development

PPD1 and PPD2 proteins share 84% identity with the TIFY transcription factor protein family (Pérez et al., 2014) and are involved in diverse protein-protein interactions, mediated by their ZIM, JAZ, and PPD-specific domains (Vanholme et al., 2007; Chini et al., 2009; Pauwels et al., 2010; Pauwels and Goossens, 2011). PPDs require other protein adaptors to form a transcriptional repressor complex, which is widely conserved among plants, other than grasses (Schneider et al., 2021, 2024).

In stomatal development, PPDs interact with KINASE-INDUCIBLE DOMAIN INTERACTING8/9 (KIX8/9) proteins to repress M ACDs (Gonzalez et al., 2015). The phenotypes of *ami-ppd*, *ppd2*, and *kix8kix9* mutants include extended M ACDs, dome-shaped leaves, and increased leaf size (Baekelandt et al., 2018; Li et al., 2018). Similar to PPDs, KIX8/9 are present in the vast majority of plants, with the exception of the *Poaceae* family (Gonzalez et al., 2015). PPD-KIX8/9 complex stability is regulated by 26S proteasome-dependent degradation controlled by STERILE APETALA (SAP/SOD3), an F-box protein that forms part of SKP1/Cullin/F-box E3 ubiquitin ligase complex, targeting KIX-PPD (Li et al., 2018). SAP physically associates with PPDs and KIX8/9. Consequently, up- or down-regulation of SAP alters M ACD and organ size (Wang et al., 2016; Li et al., 2018).

PPD2 and KIX8 have dominant roles in M ACD regulation. The phenotypes of *ppd1* and *kix9* mutants do not differ in cotyledon area to those of wild type, whereas *kix8* and *ppd2* mutants have increased areas. Further, these differences are enhanced in *kix8kix9* and *ppd1ppd2* double mutants, and even more pronounced in the quadruple mutant, *kix8kix9ppd1ppd2*; suggesting that PPD1 and KIX9 can modulate epidermal development, but require PPD2 and KIX8 to exert their effects (Liu et al., 2020).

PPDs expression and function are not restricted to stomatal lineage cells and leaf tissue, both are widely expressed and also modulate development of root, stem, inflorescence, flower, silique, and seed (Zhu et al., 2020). Hence, the complex functional plasticity of PPDs depends on the distinct molecular contexts imposed by tissue-dependent microenvironments.

## 5 PEAPOD integration of light regulation, cell cycle and stomatal development

**Figure 2:**
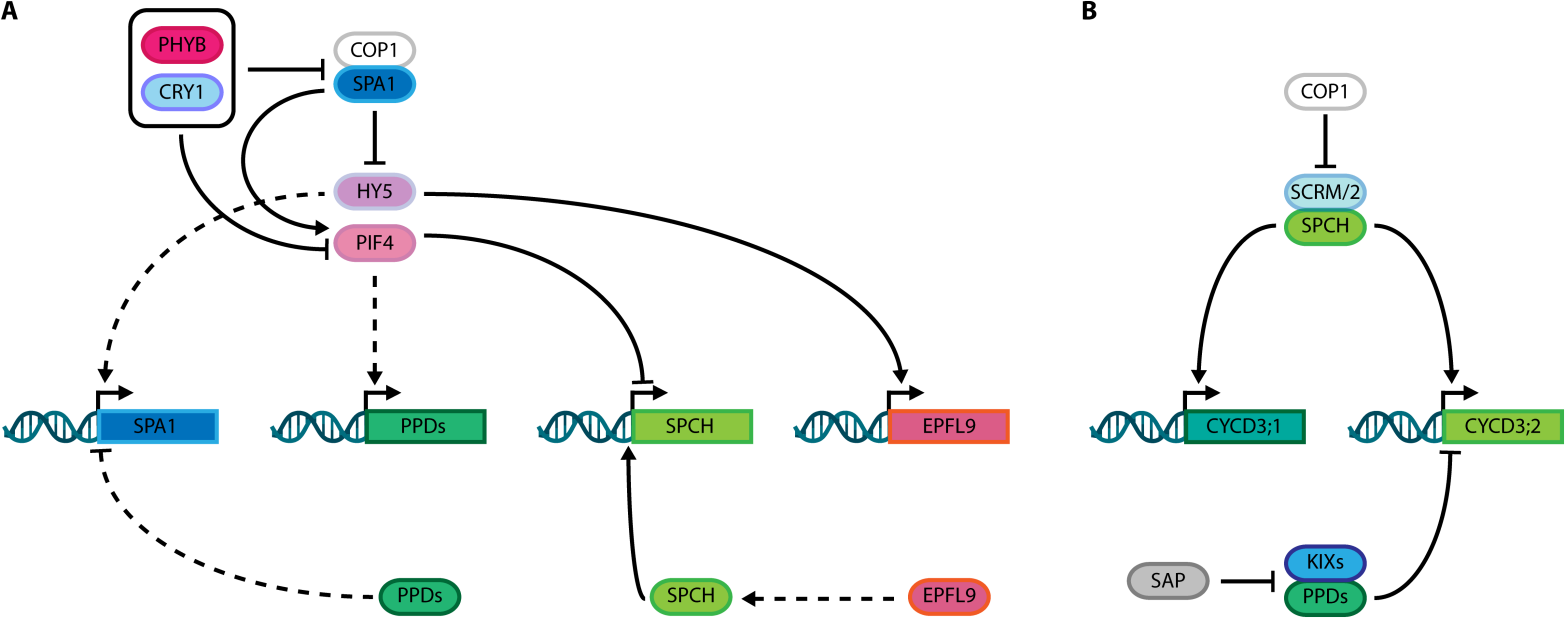
**(A)** Integration of PEAPODs (PPD) function with the control of light signaling networks comprising PHYB, CRY1, COP1, SPA1, HY5 and PIF4; and stomatal development SPCH-EPFL9 module. **(B)** Schematic overview showing interconnection of PPD complex, and its direct regulator SAP, with SPCH, SCRM/2 and the cell cycle machinery through their known targets, *CYCD3;1* and *CYCD3;2*. Dashed lines: indirect activation/stabilization or repression/degradation. Solid lines: direct activation/stabilization or repression/degradation.

PPDs are involved in light signaling, a process also linked to control of stomatal development, revealing an interplay among PPDs, stomatal genes, and key regulators of the light signaling network. Light is composed of distinct wavelengths, including the red and blue spectra, perceived by diverse plant photoreceptors. Red/far-red (FR) wavelengths are perceived by phytochromes (PHYs) (Chen and Chory, 2011), whereas blue/UV-A are sensed by cryptochromes (CRYs) (Cashmore et al., 1999). Both PHY and CRY light-receptors regulate photomorphogenesis via complex regulatory mechanisms (Jiao et al., 2007). In Arabidopsis, the PHY gene family comprises five members (PHYA to PHYE), where PHYA/B are the most prominent regulators of growth and development (Franklin and Quail, 2010). PHYA has a major role in FR perception and dark transition, whereas PHYB is important in red light detection, as it is stable under these conditions (Clough and Vierstra, 1997). The CRY gene family includes two receptors, CRY1 and CRY2, which regulate several developmental processes, including hypocotyl elongation, flowering time and stomatal development (Guo et al., 1998; Cao et al., 2021). The PHYTOCHROME-INTERACTING FACTOR (PIF) family comprises eight PIFs (PIF1–8) with distinct roles in development and modulation of light signaling, which connect both PHY and CRY light receptors (Kim et al., 2024). Amongst PIFs, PIF4 is a predominant factor that controls both light signaling and thermomorphogenesis (Xu and Zhu, 2021), with light-receptor and transduction signaling pathways partially converging on PIF4. Physical interaction between PIF4 and the active form of PHYB causes PIF4 ubiquitylation and subsequent degradation by the 26S proteasome (**Figure 2A**; Xu et al., 2015). CRY1 regulates PIF4 activity under blue light and warm-temperature, repressing its binding activity to target gene cis-regulatory elements (Ma et al., 2016; Pedmale et al., 2016).

Regarding the role of PHYs in stomatal development, only PHYB is reported to modify stomatal development via PIF4 under fluctuating light intensity (Casson et al., 2009; Casson and Hetherington, 2014). Further, PIF4 directly represses *SPCH* transcription under supra-optimal temperature conditions (Lau et al., 2018). Among CRYs, CRY1 promotes stomatal development by blue light-dependent physical interaction with SPCH, which enhances its DNA-binding activity (Cao et al., 2021; Chen et al., 2025).

Upon light perception, PHYs and CRYs inactivate two classes of repressors that act in Arabidopsis light signaling and stomatal developmental networks, including complexes comprised of CONSTITUTIVE PHOTOMORPHOGENIC 1 (COP1) and SUPPRESSOR OF PHYTOCHROME A-105 (SPA) family proteins. The *SPA* gene family includes four genes (*SPA1–4*) that positively control COP1 E3 ubiquitin ligase activity (Laubinger et al., 2004; Hoecker, 2017). These molecules constitute an important hub connecting light signaling, stomatal development, and PPD gene networks. SPA1 phosphorylates and stabilizes PIF4 (Lee et al., 2020), whereas PPDs repress *SPA1* transcription (White, 2022). Additionally, light intensity mediates indirect transcriptional activation of PPDs by PIF4 (White, 2022), while COP1 degrades SCRM/2 in a light-dependent manner, thereby modulating stomatal development (Kang et al., 2009; Lee et al., 2017). In parallel, ELONGATED HYPOCOTIL5 (HY5), a bZIP transcription factor whose accumulation promotes photomorphogenesis, is degraded in a light-dependent manner via the COP1/SPA1 complex (Saijo et al., 2003; Wang et al., 2021b). Strikingly, whereas HY5 indirectly activates *SPA1*, PPDs repress *SPA1* transcription, connecting these two pathways (**Figure 2A**). Furthermore, HY5 directly binds and controls the expression of stomatal development genes in a light-dependent way, by modulating paracrine signaling mediated by EPIDERMAL PATTERNING FACTOR LIKE9 (EPFL9/STOMAGEN), a peptide that stabilizes SPCH, which self-regulates its expression (Hunt et al., 2010; Lau et al., 2014; Wang et al., 2021a). These roles of PPDs in modulation of light signaling suggest an indirect mechanism for controlling stomatal number in response to light intensity and might indicate an effect on stomatal fate acquisition by halting M ACD in a light-dependent manner. Nonetheless, PPD regulation of stomatal development is not solely related to the crosstalk between light signaling and stomatal gene network.

The cell cycle machinery is also directly linked to cell divisions in stomatal lineages, controlling timing and cell phase states during M-GMC-GC differentiation (Desvoyes and Gutierrez, 2020; Han et al., 2022; Zuch et al., 2023; Han and Torii, 2019; Xie et al., 2010). The CYCLIN (CYC) D gene family is among regulators promoting cell division, and includes the D-3 type Cyclin (*CYCD3*) genes. The three Arabidopsis CYCD3 proteins are: CYCLIN D3;1 (CYCD3;1), CYCLIN D3;2 (CYCD3;2), and CYCLIN D3;3 (CYCD3;3), which exhibit different expression patterns, but all promote cell division in Arabidopsis tissues (Menges et al., 2006), and influence cell quantity in leaves, as demonstrated by the reduced cell numbers in the triple *cycD3;1–3* mutant (Dewitte et al., 2007). Together with KIX8/9, PPD2 directly represses *CYCD3;2* and *CYCD3;3* transcription (Gonzalez et al., 2015), while SPCH up-regulates *CYCD3;1* and *CYCD3;2* (**Figure 2B**), whose transcripts accumulate in Ms at early stages of lineage development and are associated with proliferative stages of stomatal lineages (Adrian et al., 2015; Vatén et al., 2018). Notably, *CYCD3;2* is the only D-3 type Cyclin both directly bound and up-regulated by SPCH, and also found to be upregulated in the *ami-ppd* RNAseq dataset (Lau et al., 2014; Gonzalez et al., 2015). Further, *CYCD3;2* overexpression phenocopies the dome-shaped leaves observed in *ppd*, *ami-ppd*, and *ppd2* mutants, without altering leaf size. Conversely, *CYCD3;1* and *CYCD3;2* inactivation partially restores the *ami-ppd* phenotype (Baekelandt et al., 2018). Moreover, as repression of *SPA1* transcription by PPDs influences the HY5-EPFL9 module, PPDs function might be affecting SPCH activity indirectly. This could putatively represent a mechanism of indirect modulation of stomatal development by light signaling via PPD-mediated cell cycle control.

## 6 Conclusions and perspectives

In summary, the PPD complex acts as a molecular hub, integrating both light signaling and CYCD3-mediated cell cycle control, while restricting M self-renewing activity. Hence, stomatal phenotypes of PPD complex mutants may be partially explained by the crosstalk among these distinct hubs, although a direct connection between PPDs and stomatal development remains to be established. Additionally, the role of PPDs and their adaptor proteins KIX8/9 in limiting the M ACDs requires additional investigation, as *ppd* stomatal phenotypes have not been mechanistically explained. Further studies are also required to explore potential new roles of the PPD complex in hormonal and environmental regulation of stomatal development. Given the importance of stomatal development in leaf morphogenesis and the regulatory role of the PPD complex in organ growth, deepening our knowledge in this area may be instrumental for improving crop productivity through translational approaches.
